# Acute Posterior Multifocal Placoid Pigment Epitheliopathy Presenting with Multiple Serous Retinal and Bacillary Layer Detachments: A Diagnostic Challenge

**DOI:** 10.22336/rjo.2026.42

**Published:** 2026

**Authors:** Vipin Rana, Utkarsh Roodkee, Ranjit Goenka, Raihan Mondal, Mohit Dogra, Ashish Markan, Atul Arora, Jaya Kuashik

**Affiliations:** 1 Department of Ophthalmology, Command Hospital (Eastern Command), Kolkata, India; 2 Advanced Eye Center, Post Graduate Institute of Medical Education Research, Chandigarh, India; 3 Dr. RP Center, All India Institute of Medical Education and Research, New Delhi, India; 4 Department of Ophthalmology, MH Panagarh (Eastern Command), Kolkata, India

**Keywords:** APMPPE, bacillary layer detachment, serous retinal detachment, Vogt-Koyanagi-Harada syndrome, multimodal imaging, APMPPE = Acute Posterior Multifocal Placoid Pigment Epitheliopathy, BALAD = Bacillary Layer Detachment, BCVA = Best-Corrected Visual Acuity, CNS = Central Nervous System, FFA = Fundus Fluorescein Angiography, HRCT = High-Resolution Computed Tomography, ICG = Indocyanine Green Angiography, OCT = Optical Coherence Tomography, OCTA = Optical Coherence Tomography Angiography, EDI-OCT = Enhanced Depth Imaging Optical Coherence Tomography, SRD = Serous Retinal Detachment, TPHA = Treponema Pallidum Hemagglutination Assay, VDRL = Venereal Disease Research Laboratory, VKH = Vogt-Koyanagi-Harada

## Abstract

**Purpose:**

To describe a diagnostically challenging case of Acute Posterior Multifocal Placoid Pigment Epitheliopathy (APMPPE) in a young girl, presenting with features mimicking Vogt-Koyanagi-Harada (VKH) syndrome.

**Methods:**

An 18-year-old Asian Indian girl presented with sudden bilateral visual diminution and metamorphopsia for three days. Clinical examination and multimodal imaging were performed, including Enhanced Depth Imaging Optical Coherence Tomography (EDI-OCT), Fundus Fluorescein Angiography (FFA), Indocyanine Green Angiography (ICG), and Optical Coherence Tomography Angiography (OCTA). Systemic evaluation included the Mantoux test, QuantiFERON-TB Gold, HRCT of the chest, abdominal ultrasonography, and routine blood work.

**Results:**

BCVA was 20/40 in both eyes. Fundus examination revealed mild vitritis, multiple serous retinal detachments (SRD), and yellowish placoid lesions at the posterior pole. EDI-OCT showed SRD with bacillary layer detachment and mild choroidal thickening in both eyes. FFA revealed early hypofluorescence with late staining; ICG showed persistent hypocyanescent lesions localized to the posterior pole. OCTA demonstrated choriocapillaris flow voids. Mantoux and QuantiFERON-TB Gold were strongly positive; other systemic investigations were unremarkable. Given the absence of VKH prodromal features and features on multimodal imaging, a diagnosis of APMPPE was made. The patient was observed without corticosteroid therapy, and complete resolution of SRD and bacillary layer detachment occurred within 1 week.

**Discussion:**

The initial presentation with bilateral SRDs and BALADs strongly suggested VKH syndrome. However, the absence of systemic prodromal symptoms, combined with characteristic angiographic features, led to a diagnosis of APMPPE. The rapid, spontaneous resolution of these atypical detachments without corticosteroid therapy further confirmed the self-limiting nature of APMPPE, in sharp contrast to the prompt immunosuppression typically required for VKH.

**Conclusion:**

APMPPE can rarely present with atypical clinical features such as SRD and BALAD. Clinicians must integrate multimodal imaging with systemic evaluations to accurately differentiate it from VKH syndrome, thereby preventing misdiagnosis and avoiding unnecessary treatment.

## Introduction

Acute posterior multifocal placoid pigment epitheliopathy (APMPPE), a form of White Dot Syndrome, was initially identified by Gass in 1968 as an inflammatory chorioretinopathy that predominantly affects healthy young adults and typically follows a benign, self-limiting course [1]. Clinically, it is characterized by the sudden onset of bilateral visual symptoms and multiple placoid lesions at the posterior pole. While its classic presentation is well described, atypical features such as multiple serous retinal detachments (SRDs) and bacillary layer detachments (BALADs) are rarely observed. They may cause considerable diagnostic confusion, particularly in conditions such as Vogt-Koyanagi-Harada (VKH) syndrome [2].

We report a diagnostically challenging case of APMPPE in a young female who presented with bilateral SRD and BALAD, findings more typically associated with VKH. This case underscores the importance of recognizing uncommon manifestations of APMPPE and integrating multimodal imaging and systemic clues to avoid misdiagnosis and overtreatment.

## Case report

**Fig. 1:**
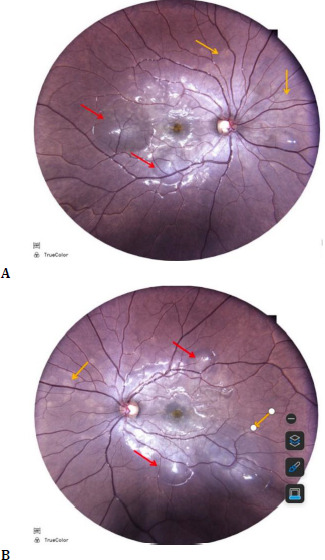
A, B Ultra-widefield true-color fundus photographs of both eyes reveal multiple dome-shaped elevations at the posterior pole, varying in size from approximately half a disc diameter to four-disc diameters, consistent with serous retinal detachments (red arrows). Additionally, several flat, round-to-placoid, yellowish lesions with ill-defined margins are scattered across the posterior pole, ranging in size from one-fourth to one disc diameter (yellow arrows), suggestive of inflammatory retinal pigment epithelial involvement

An 18-year-old Asian Indian girl presented with a sudden onset of painless diminution of vision in both eyes, accompanied by metamorphopsia for the past three days. There was no history of fever, tinnitus, or other viral prodromal symptoms. On examination, the best-corrected visual acuity (BCVA) was 20/40 in both eyes. Intraocular pressures were within normal limits. Anterior segment examination was unremarkable in both eyes. Posterior segment evaluation revealed mild vitritis and multiple SRD confined to the posterior pole in both eyes. Additionally, multiple yellowish placoid lesions were noted at the posterior pole, varying in size from approximately one-fourth disc diameter to one disc diameter (Fig. 1A, B). Based on these findings, a differential diagnosis of VKH syndrome and APMPPE was considered.

**Fig. 2:**
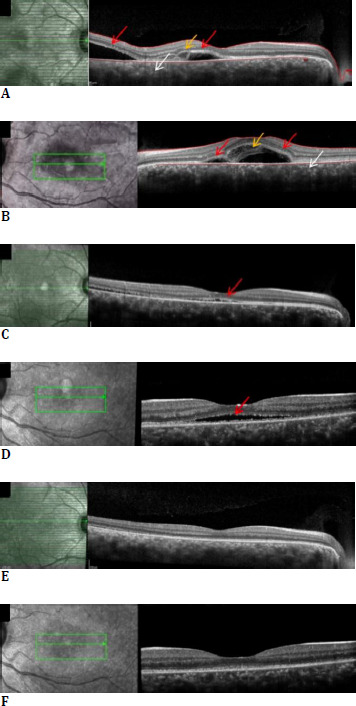
Enhanced depth imaging-optical coherence tomography (EDI-OCT) of the macula in both eyes. A, B: Baseline EDI-OCT shows multiple areas of serous retinal detachment (red arrows) along with bacillary layer detachment (BALAD) involving the fovea (yellow arrows). There is also evidence of mildly increased choroidal thickness in both eyes (white arrows). C, D: Follow-up Day 4 EDI-OCT shows spontaneous resolution of the serous retinal detachment in both eyes. E, F: Subsequent Day 7 EDI-OCT demonstrates complete resolution of the serous retinal detachment and restoration of the outer retinal layers in both eyes

**Fig. 3:**
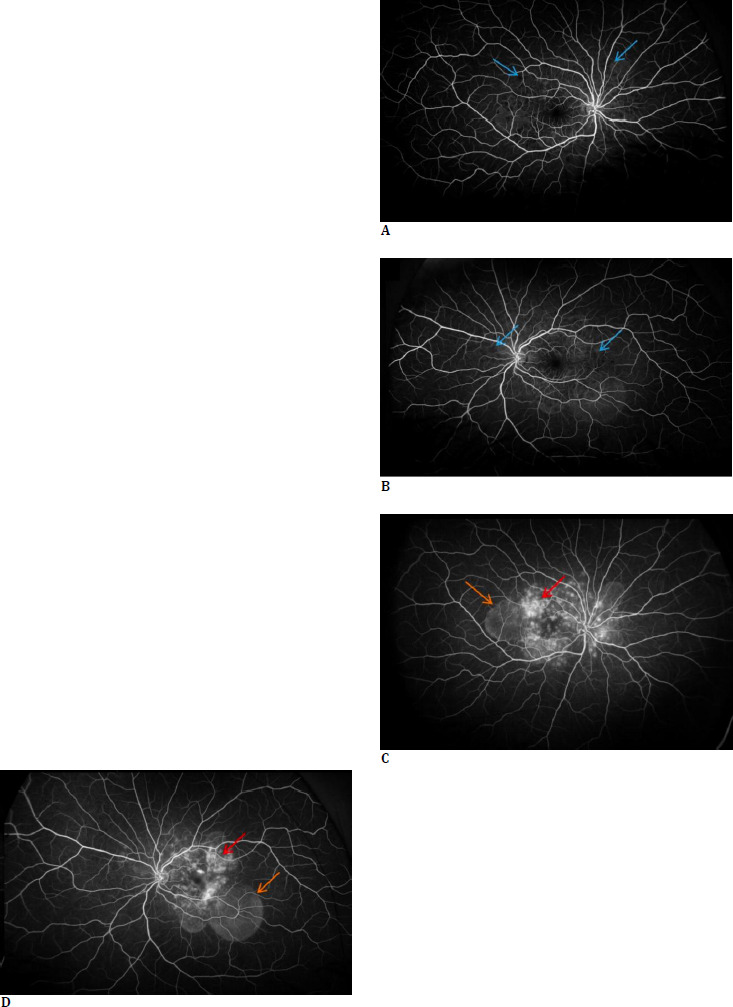
A, B Venous phase fundus fluorescein angiography (FFA) of both eyes shows multiple hypofluorescent spots in the posterior pole (blue arrows). C, D Late-phase FFA of both eyes demonstrates staining and increased hyperfluorescence of the lesions (red arrows), along with pooling of dye in the regions of serous retinal detachment (orange arrows)

**Fig. 4:**
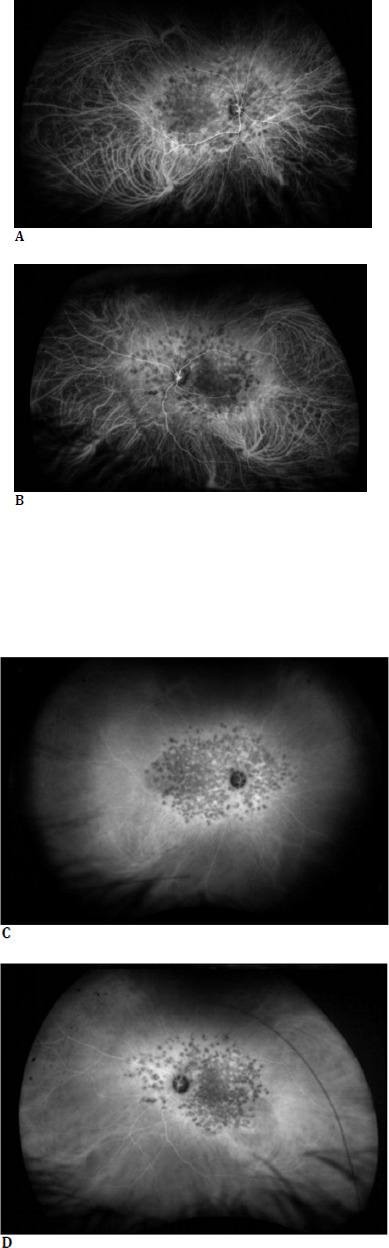
A, B Early phases of Indocyanine green angiography (ICG) of both eyes demonstrated multiple hypocyanescent dots scattered throughout the posterior pole, indicating areas of choriocapillaris non-perfusion. C, D Late phase of ICG of both eyes reveals persistent hypocyanescence in the same areas

Enhanced Depth Imaging Optical Coherence Tomography (EDI-OCT) revealed multiple areas of SRD with BALAD in the fovea bilaterally. Choroidal thickness was mildly increased (Fig. 2A, B). Fundus fluorescein angiography (FFA) showed early hypofluorescent dots, followed by hyperfluorescence in the late phase, in both eyes (Fig. 3A-D). Indocyanine green angiography (ICG) demonstrated multiple hypocyanescent spots in both eyes that persisted from early to late phases and were confined to the posterior pole (Fig. 4A-D). Optical Coherence Tomography Angiography (OCTA) revealed flow voids in the choriocapillaris layer corresponding to the placoid lesions.

Systemic investigations showed a strongly positive Mantoux test (22 × 26 mm induration) and a positive QuantiFERON-TB Gold test, suggestive of latent tuberculosis infection. However, high-resolution computed tomography (HRCT) of the chest and abdominal ultrasonography were unremarkable, with no evidence of active tuberculosis or sarcoidosis. Additional laboratory tests, including complete blood count, VDRL, TPHA, and viral serologies, were within normal limits.

Considering the absence of systemic VKH features, the localized posterior segment involvement, and characteristic angiographic patterns, a diagnosis of APMPPE was made. The patient was managed conservatively without systemic corticosteroids. On follow-up, there was complete resolution of subretinal fluid in both eyes within one week. The placoid lesions faded entirely over the following two weeks, with complete resolution of visual symptoms, improvement of BCVA to 20/20 in both eyes, and restoration of outer retinal architecture as confirmed on OCT. A neurology consultation was obtained to rule out CNS vasculitis.

## Discussion

The clinical presentation of our patient resembled both APMPPE and VKH syndrome, highlighting the diagnostic complexity that can arise when atypical features are encountered.

In particular, the presence of bilateral SRD and BALAD, more commonly described in VKH, initially raised concern for that diagnosis. However, the absence of systemic prodromal signs such as meningism, tinnitus, or integumentary changes, combined with quiet anterior segments and mild vitritis, prompted reconsideration.

The clinical findings ultimately aligned more closely with APMPPE. Although Gass first described SRD in 1968 as a self-limiting inflammatory chorioretinopathy, it was not included in the original description of APMPPE. While SRD remains an uncommon feature of APMPPE, a few cases have reported its presence [1-6]. Additionally, our case demonstrated BALAD on OCT, a rare but documented finding. Kohli et al. reported three such cases that resolved spontaneously within a week [4]. Similar to their series, the BALAD in our case also resolved within one week. However, unlike their cases, which involved only unilateral eyes and did not report multiple SRDs, our patient presented with bilateral involvement and multiple SRDs. Verma et al. also reported two similar cases of APMPPE with BALAD accompanied by SRD [7]. However, in contrast to our case, in which no treatment was administered, their patients received oral prednisolone at 1 mg/kg. In our case, the SRD resolved spontaneously within 7 days without treatment, underscoring the benign, self-limited course of the disease. We speculated that the rapid resolution of SRD might contribute to its under-recognition in APMPPE.

Multimodal imaging findings further supported the diagnosis. FFA revealed early hypofluorescent and late staining lesions, characteristic of APMPPE. ICG showed multiple hypocyanescent spots that persisted from the early to the late phases and were localized to the posterior pole, unlike VKH, which typically shows widespread hypocyanescence. OCTA demonstrated flow voids at the level of the choriocapillaris corresponding to placoid lesions, likely reflecting hypoperfusion rather than signal blockage [8].

Systemic investigations revealed a strongly positive Mantoux test and QuantiFERON-TB Gold assay, indicative of latent tuberculosis infection. APMPPE has been associated with several systemic conditions, including tuberculosis, sarcoidosis, Bartonella infection, psoriasis, and post-vaccination states (e.g., hepatitis B, varicella, tetanus, and polio) [9]. Given the potential for APMPPE to be associated with cerebral vasculitis, neurological consultation was undertaken to rule out subclinical CNS involvement.

The natural course of our patient, characterized by spontaneous resolution of SRD and improvement in vision without corticosteroid therapy, was a key differentiator from VKH, which typically requires prompt and prolonged immunosuppression [10]. Recognizing this self-limiting trajectory is essential to avoid unnecessary treatment.

## Conclusion

This case underscores the importance of correlating clinical features, imaging findings, and systemic work-up to differentiate APMPPE from other posterior uveitic entities. While features like SRD and BALAD may initially suggest VKH, their presence does not preclude a diagnosis of APMPPE. Awareness of such atypical imaging findings is critical to making an accurate diagnosis and guiding appropriate, conservative management.

## References

[1] Gass JD (2003). Acute posterior multifocal placoid pigment epitheliopathy 1968. Retina.

[2] Sharma S, Kharel R, Parajuli S, Karki P, Joshi SN (2023). Role of multimodal imaging in differentiating unilateral APMPPE from unilateral Harada disease-a case report. Ann Med Surg (Lond).

[3] Mahjoub A, Ben Abdesslem N, Ben Youssef C, Zaafrane N, Mahjoub A, Ben Abderrazek A, Sellem I, Chtioui H, Ghorbel M, Mahjoub H (2022). Acute posterior multifocal placoid pigment epitheliopathy associated with serous retinal detachment: A case report. Ann Med Surg (Lond).

[4] Kohli GM, Bhatia P, Shenoy P, Sen A, Gupta A (2022). Bacillary Layer Detachment in Hyper-acute Stage of Acute Posterior Multifocal Placoid Pigment Epitheliopathy: A Case Series. Ocul Immunol Inflamm.

[5] Lee GE, Lee BW, Rao NA, Fawzi AA (2011). Spectral domain optical coherence tomography and autofluorescence in a case of acute posterior multifocal placoid pigment epitheliopathy mimicking Vogt-Koyanagi-Harada disease: case report and review of literature. Ocul Immunol Inflamm.

[6] Su G, Meng J, Li H, Cai SJ (2022). Multimodal imaging of the course of retinal changes in acute posterior multifocal placoid pigment epitheliopathy with bilateral retinal detachment: a case report. BMC Ophthalmol.

[7] Lalit V, Shweta S, Avnindra G, Bhanupriya Vijay K (2025). Subfoveal bacillary layer detachment peculiar in the early stage of acute posterior multifocal placoid pigment epitheliopathy (APMPPE). Indian Journal of Ophthalmology-Case Reports.

[8] Kinouchi R, Nishikawa N, Ishibazawa A, Yoshida A (2017). Vascular rarefaction at the choriocapillaris in acute posterior multifocal placoid pigment epitheliopathy viewed on OCT angiography. Int Ophthalmol.

[9] Saleh M (2020). Placoid pigment epitheliopathy and serpiginous choroiditis. J Fr Ophtalmol.

[10] Papasavvas I, Mantovani A, Herbort CP (2022). Acute Posterior Multifocal Placoid Pigment Epitheliopathy (APMPPE): A Comprehensive Approach and Case Series: Systemic Corticosteroid Therapy Is Necessary in a Large Proportion of Cases. Medicina (Kaunas).

